# High SNR Acquisitions Improve the Repeatability of Liver Fat Quantification Using Confounder-corrected Chemical Shift-encoded MR Imaging

**DOI:** 10.2463/mrms.mp.2016-0081

**Published:** 2017-02-13

**Authors:** Utaroh Motosugi, Diego Hernando, Curtis Wiens, Peter Bannas, Scott. B Reeder

**Affiliations:** 1 Department of Radiology, University of Wisconsin, Madison, WI, USA; 2 Department of Radiology, University of Yamanashi, 1110 Shimokato, Chuo, Yamanashi 409-3898, Japan; 3 Department of Radiology, University Hospital Hamburg-Eppendorf, Hamburg, Germany; 4 Department of Biomedical Engineering, University of Wisconsin, Madison, WI, USA; 5 Department of Medical Physics, University of Wisconsin, Madison, WI, USA; 6 Department of Medicine, University of Wisconsin, Madison, WI, USA; 7 Department of Emergency Medicine, University of Wisconsin, Madison, WI, USA

**Keywords:** chemical shift-encoded magnetic resonance imaging, proton density fat fraction, fatty liver disease, signal to noise ratio, repeatability

## Abstract

**Purpose:**

To determine whether high signal-to-noise ratio (SNR) acquisitions improve the repeatability of liver proton density fat fraction (PDFF) measurements using confounder-corrected chemical shift-encoded magnetic resonance (MR) imaging (CSE-MRI).

**Materials and Methods:**

Eleven fat-water phantoms were scanned with 8 different protocols with varying SNR. After repositioning the phantoms, the same scans were repeated to evaluate the test-retest repeatability. Next, an in vivo study was performed with 20 volunteers and 28 patients scheduled for liver magnetic resonance imaging (MRI). Two CSE-MRI protocols with standard- and high-SNR were repeated to assess test-retest repeatability. MR spectroscopy (MRS)-based PDFF was acquired as a standard of reference. The standard deviation (SD) of the difference (Δ) of PDFF measured in the two repeated scans was defined to ascertain repeatability. The correlation between PDFF of CSE-MRI and MRS was calculated to assess accuracy. The SD of Δ and correlation coefficients of the two protocols (standard- and high-SNR) were compared using *F*-test and *t*-test, respectively. Two reconstruction algorithms (complex-based and magnitude-based) were used for both the phantom and *in vivo* experiments.

**Results:**

The phantom study demonstrated that higher SNR improved the repeatability for both complex- and magnitude-based reconstruction. Similarly, the *in vivo* study demonstrated that the repeatability of the high-SNR protocol (SD of Δ = 0.53 for complex- and = 0.85 for magnitude-based fit) was significantly higher than using the standard-SNR protocol (0.77 for complex, *P* < 0.001; and 0.94 for magnitude-based fit, *P* = 0.003). No significant difference was observed in the accuracy between standard- and high-SNR protocols.

**Conclusion:**

Higher SNR improves the repeatability of fat quantification using confounder-corrected CSE-MRI.

## Introduction

Hepatic steatosis is the abnormal accumulation of intracellular fat in hepatocytes, primarily in the form of triglycerides. Similar to alcoholic fatty liver disease, nonalcoholic fatty liver disease (NAFLD) can progress to inflammation and fibrosis, eventually resulting in cirrhosis.^1^ Fortunately, with intervention, steatosis is reversible, and reduction in liver fat may diminish many of its associated risks.^2^

An accurate and precise (i.e. repeatable) method to detect and monitor hepatic steatosis is urgently needed for the management of patients with NAFLD. Non-targeted percutaneous liver biopsy is the current reference standard to detect hepatic steatosis and definitely diagnose NAFLD.^3,4^ However, biopsy is invasive, expensive, and unsuitable for longitudinal treatment monitoring.^5,6^ Further, biopsy suffers from high variability for quantitative assessment of liver disease, including steatosis.^7,8^ Hence, alternative methods have been proposed for liver fat quantification, including ultrasound-^9^ and computed tomography-based methods,^10,11^ and magnetic resonance imaging (MRI)-based chemical shift-encoded (CSE) methods.^12–14^

Recently, MRI is increasingly used for the evaluation of the liver either for focal liver lesion and diffuse liver disease.^15^ Combined with other imaging techniques including hepatobiliary contrast agents for assessment of focal liver lesions^16,17^ and magnetic resonance (MR) elastography for assessment of liver fibrosis,^18^ liver fat quantification using CSE-MRI^19^ contributes to the comprehensive assessment of liver disease.^20^ By addressing all relevant confounding factors,^21–24^ CSE-MRI provides quantitative maps of proton-density fat-fraction (PDFF), a well-validated biomarker of triglyceride concentration, over the entire liver.^25–27^ As has been demonstrated in multiple recent studies, CSE-MRI provides accurate and reproducible liver PDFF quantification across different vendors and field strengths,^28,29^ over a broad range of PDFF (e.g. 0–50%).

However, recent studies suggest the need for accurate and precise PDFF quantification at low fat-fractions. In the Dallas-Heart study,^30^ a fat-fraction of 5.56% was established as the 95% threshold in a patient population with no identifiable risk factors for steatosis. Tang, et al. suggested the cutoff value of 6.5% in PDFF to discriminate steatosis grade ≥1 from grade 0.^31^ Another recent study identified a threshold of 4.96% for indicating substantial macrovesicular steatosis in pathological specimens.^32^ More recently, an even lower threshold (PDFF = 3.5%) was shown to be highly predictive of metabolic syndrome in adolescent females.^26^

Reliable PDFF quantification near these low thresholds requires highly accurate and precise techniques. Previous studies established the repeatability (95% confidence interval) of CSE-MRI as ±2–4 percent point (pp) at 1.5T and 3T,^28^ or ±1–2 pp using CSE-MRI with breath hold and respiratory-triggering methods.^33^ These relatively broad confidence intervals may limit the utility of CSE-MRI for fat quantification over the low PDFF range (e.g. 0–10%).

Sufficient signal-to-noise ratio (SNR) is a key factor required for repeatable measurements. SNR has a direct relationship with voxel size. In general, the voxel size of standard CSE-MRI acquisitions is ∼10–37 mm^3^ at 1.5T.^32,34^ High spatial resolution is required to identify anatomical or pathological objects, e.g. vessels or lesions, for accurate PDFF/ ${\text{R}}_{2}^{*}$ measurements by avoiding partial volume effects. In contrast, high spatial resolution limits the SNR of the acquired images. However, high spatial resolution may not be needed for assessment of diffuse liver disease (i.e. NAFLD), particularly given the relatively large regions of interest (ROIs) used with PDFF maps in the liver.^35^ Therefore, we hypothesize that increasing SNR through small reductions in spatial resolution may improve the test-retest repeatability of CSE-MRI without affecting its accuracy.

Hence, the purpose of this study was to demonstrate the relationship of increased SNR and improved repeatability of CSE-MRI-based PDFF measurements. The relationship between SNR and repeatability was evaluated using theoretical analysis, fat-water phantom imaging, as well as *in vivo* liver imaging.

## Materials and Methods

### Theoretical estimation of repeatability

Test-retest repeatability (standard deviation between repeated acquisitions) was estimated based on Cramér-Rao Bound (CRB) calculations.^36,37^ For this estimation, we assumed the following imaging parameters for CSE-MRI: 1.5T, 6 acquired echoes acquired with minimum TE of 1.23 ms with echo spacing of 1.88 ms (same as in the phantom acquisitions; see below), a multi-peak fat signal model with six peaks,^38^ true PDFF = 5%. In these calculations, voxel-wise echo image SNR (measured as the SNR at a theoretical TE = 0 ms image) was varied from 5 to 300 to assess the relationship between SNR and repeatability. For these calculations, PDFF measurements were considered over an ROI consisting of 117 voxels (as in the phantom experiments; see below). CRB-based calculations of repeatability (standard deviation of the difference between ROI-based measurements of PDFF) were calculated as $\sqrt{\frac{2}{\text{ROI}\_\text{Size}}}{\mathrm{SD}}_{\mathrm{PDEF}}$ , where *SD_PDFF_* is the theoretical standard deviation of voxel-wise PDFF measurements obtained at each SNR level, measured both for complex-fitting and for magnitude-fitting based PDFF quantification.

## Phantom Experiments

### Phantom construction and setup

A fat-water phantom was constructed, consisting of multiple vials with agar based emulsions of peanut oil and water, similar to previous works.^39^ The phantom was comprised of 11 cylindrical glass vials (outer diameter = 28 mm, height = 98 mm) with oil-water emulsions including nominal fat fraction (FF) of 0–10% by 1% (11 phantoms in total). Each vial contained 40 mL of oil-water emulsion, where the water component included agar (2% w/v) to form the gel, CuSO_4_ (3 mM) to shorten the T_1_ of water, sodium dodecyl sulfate (43 mM) as surfactant, NaCl (43 mM) to adjust the conductivity, and sodium benzoate (3 MM) as preservative.

### Imaging phantoms

Imaging was performed on a clinical 1.5T scanner (Signa HDxt, GE Healthcare, Waukesha, WI) with an 8-channel body coil. The CSE-MRI acquisitions were performed repeatedly using a three dimensional (3D)-spoiled gradient echo acquisition, with eight different protocols by changing the spatial resolutions and number of excitations (NEXs). After repositioning the phantoms, the same scans were repeated to evaluate test-retest repeatability. MR parameters were designed with three protocols with varying spatial resolution:

1. High spatial resolution with low SNR, with voxel size of 1.6 × 1.6 × 1.5 mm, partial *k_y_* acquisition of 30%, bandwidth of ±167 kHz.
2. Intermediate spatial resolution with intermediate SNR, acquired with identical parameters except 1.9 × 1.9 × 4.0 mm voxel size and bandwidth of ±91 kHz.
3. Low spatial resolution with high SNR, acquired with identical parameters except 2.5 × 2.5 × 6.0 mm voxel size and bandwidth of ±63 kHz.

High and medium resolution protocols were scanned with 1, 4, and 16 signal averages (NSA), and low resolution protocols with 1 and 4 NSA. Other acquisition parameters included: repetition time (TR) of 21.2 ms; maximum and minimum echo time (TE) of 10.5–10.7 ms and 1.1–1.2 ms (six echoes in total); 32 slices; 36 cm field of view; five degree flip angle. Parallel imaging was not used for the phantom experiments.

The SNR of the eight different protocols were estimated by the following equation:

$$\mathrm{SNR}=\frac{\mathrm{mean}\;(\mathrm{abs}(S))}{\mathrm{std}\;(\mathrm{real}(S'))}$$

where *S* are the complex signal intensities within the ROI of the FF = 0% phantom and *S’* is the signal from real channel of the background noise outside the vials, to avoid bias related to Rician noise distribution.^40,41^

## In vivo Study

### Subjects

This prospective *in vivo* study was performed after obtaining approval from our local Institutional Review Board. Written informed consent was obtained from all subjects. 20 healthy subjects (mean [range] age of 30 [24–58] years; 16 men and 4 women) were prospectively recruited. Further, 28 patients (mean [range] age of 55 [19–93] years; 10 men and 18 women) who were scheduled for routine clinical abdominal MRI were also recruited prospectively. Exclusion criteria included any contraindication to MRI and age less than 18 years.

### MR imaging and spectroscopy acquisition

All imaging was performed on a clinical 1.5T scanner (Optima MR450w or Signa HDxt, GE Healthcare, Waukesha, WI) with an 8 or 12 channel phased array coil. The following three quantitative acquisitions were performed (see Table 1 for details); (a) CSE-MRI fat quantification method with standard SNR protocol (standard spatial resolution); (b) CSE-MRI with high SNR protocol (low spatial resolution); (c) single voxel multi-echo T_2_-corrected STEAM spectroscopy (MRS) as the reference standard for PDFF. The first two acquisitions (a and b) were repeated after removing the subject from the scanner bore, removing the anterior coil elements, sitting the subject up on the table, allowing the subject them lie down again and replacing the coil. Every sequence was acquired during a single breath hold (19–26 s). The entire liver was imaged using a 3D volume oriented in the axial plane for both the standard and high SNR protocols. All data were reconstructed with both complex- and magnitude-based fitting as we did for phantom study.

On one healthy volunteer, estimates of the SNR of the standard and high SNR protocols were performed using a Monte-Carlo based pseudo-multiple replica method.^42^ For these acquisitions, an additional noise-only scan was required using the same bandwidths and amplifier gains as the corresponding CSE-MRI. ROIs were drawn on the right lobe of the liver on the SNR maps to estimate the SNR.

In order to obtain a reference measurement for PDFF, Stimulated Echo Acquisition Mode (STEAM) MR spectroscopy (MRS) acquisitions were obtained over a single 2.0 × 2.0 × 2.0 cm^3^ voxel placed in the posterior lobe of the liver, avoiding major blood vessels and bile ducts. STEAM was used to minimize the effects of J-coupling.^43^ STEAM-MRS parameters included: multiple TEs = 10, 15, 20, 25, 30 ms to enable T_2_ correction, TR = 3500 ms to minimize T_1_ bias, 1 signal average, 2048 points, and a spectral width of ±2.5 kHz, acquired in 21 seconds of a single breath-hold.

### MRI- and MRS-PDFF measurements

All data were reconstructed using both complex and magnitude based water-fat reconstructions to produce quantitative PDFF maps over the entire liver.^37^ In both cases, the confounding factors of ${\text{T}}_{2}^{*}$ ,^23^ accurate spectral modeling of fat^22^ and correction for eddy currents^44^ were addressed. Low flip angles (2–5 degree) were used to minimize T_1_ bias.^21^

For phantom experiments, PDFF measurements were obtained by placing a circular region of interests containing ∼420 mm^2^ in the center of each of the 11 phantoms.

For *in vivo* study, a radiologist with 13 years’ experience in liver imaging placed ROIs in the posterior segment of the right lobe of the liver. A circular ROI was placed in the liver on PDFF maps from CSE-MRI and was co-registered with the MRS voxel as closely as possible. During the ROI placement, special attention was paid to avoid partial volume effects at the liver edge, obvious artifacts or contamination of large vessels. ROI placement was manually adjusted if any obvious image artifacts such as ghosting were identified at that location.

Fat-quantification from MRS data was performed using an in-house fitting routine that accounts for the spectral complexity of the fat signal as well as for T_2_ decay across spectra at different TEs.^45^

## Statistical Analyses

To evaluate the test-retest repeatability, we first defined the difference (Δ) as the difference of PDFF measured between test and retest acquisitions. Here we assumed that ideal mean of Δ would be 0 and standard deviation of Δ would estimate the variability observed in repeated measurements, i.e. lower standard deviation of the Δ represents higher precision (improved repeatability).

The results of repeatability (standard deviation of the Δ) in phantom scans were plotted in single logarithmic graph against SNR. In the *in vivo* study, the repeatability (standard deviation of Δ) of CSE-MRI with the standard SNR protocol was compared with the high SNR protocol using *F*-test.

To evaluate the accuracy of PDFF measurement, linear regression analysis was performed. The correlation coefficients (r) of PDFF with CSE-MRI and MRS were calculated and compared by *t*-test after Fisher’s r-z transformation.

The correlation coefficients were interpreted as no correlation for 0–0.20, fair correlation for 0.21–0.40, moderate correlation for 0.41–0.70, substantial for 0.71–0.90, and strong correlation for 0.91–1.0. A *P*-value of <0.05 was considered to be statistically significant. Statistical analyses were performed using MedCalc (MedCalc Software, Ostend, Belgium) and R version 3.1.1 (R Foundation Statistic Computing).

## Results

### Repeatability of theoretical estimation and phantom study

**Fig 1.**
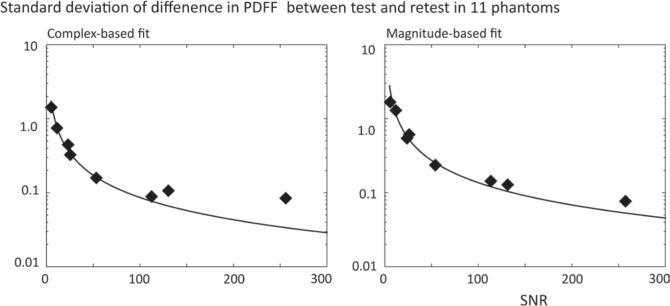
Phantom study showed test-retest repeatability improved by increasing signal-to-noise ratio (SNR) (dots) for both complex-based fitting and magnitude-based fitting. The measured standard deviation from phantom study was well matched with theoretical values (solid lines). PDFF, proton density fat fraction.

The results of repeatability (standard deviation of Δ) in phantom study were shown in Fig. 1, in which logarithmic scale of standard deviation in y-axis and SNRs for eight acquisitions in x-axis (Fig. 1). Theoretical estimations of repeatability (standard deviation) were overlaid with solid line. There was a clear and direct relationship between higher SNR and higher repeatability (lower standard deviation) obtained for both complex- and magnitude-based fits. Phantom study showed that standard deviations of Δ were decreased as SNR increase; 1.46, 0.16, and 0.09 pp for SNR of 6.0, 54.4, and 113.6, respectively in complex-based fit; 1.73, 0.24, and 0.15 pp for SNR of 6.0, 54.4, and 113.6, respectively, in magnitude-based fit.

### Repeatability and accuracy in the in vivo study

SNR estimation from the Monte-Carlo based pseudo-multiple replica method revealed that high SNR protocol of CSE-MRI had ∼3.3 times more SNR than standard SNR protocol at scanner 1 and ∼2.7 times at scanner 2 (Table 1).

**Fig 2.**
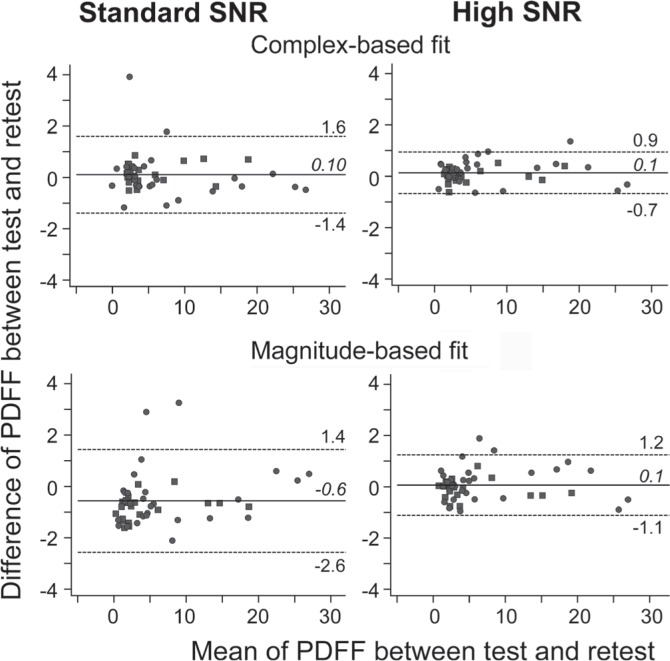
Bland-Altman plots of *in vivo* fat quantification demonstrate improved test-retest repeatability using the high signal-to-noise ratio (SNR) protocol for both complex- and magnitude-based fitting. The numbers and horizontal lines show the mean (italic and solid line) and 95^th^ percentile confidence intervals (1.96 × standard deviations (non-italic and dotted line) of Δ). PDFF, proton density fat fraction; Δ, the difference.

As shown in Fig. 2, Bland-Altman analysis demonstrates that the Δ is distributed around 0 (−0.6 to 0.1) for both standard and high SNR protocols. The standard deviation of Δ was significantly smaller for high SNR protocol (0.37 pp for complex- and 0.59 pp for magnitude-based fit) than in standard SNR protocol (0.77 pp for complex-, *P* < 0.001; and 0.94 pp for magnitude-based fit, *P* = 0.003) (Fig. 2 and Table 2).

**Fig 3.**
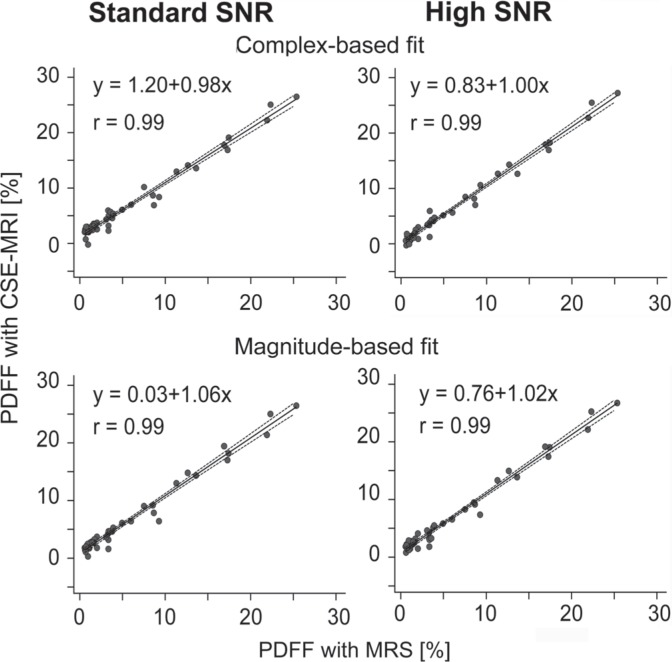
Proton density fat fraction (PDFF) measured by chemical shift-encoded magnetic resonance imaging (CSE-MRI) were well correlated with PDFF measured by magnetic resonance spectroscopy (MRS) for either standard or high signal-to-noise ratio (SNR) protocols. Correlation lines are shown with 95% confidence intervals.

**Fig 4.**
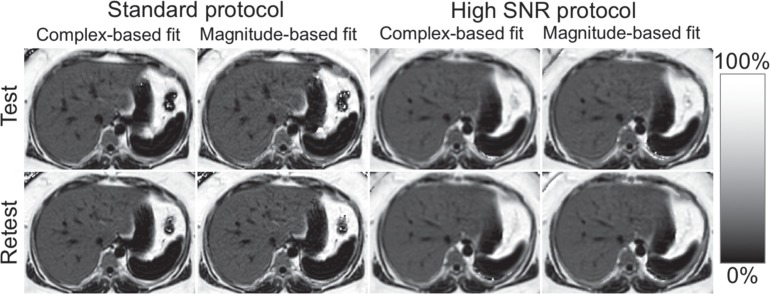
Examples of proton density fat fraction (PDFF) maps of standard and high signal-to-noise ratio (SNR) protocols for test and retest scans. All anatomical details including intrahepatic vessels were preserved in the high SNR protocols compared with standard protocols.

Correlation between the PDFF of MRS and CSE-MRI were very strong in both standard and high SNR protocols (*r* = 0.985–0.987). (Figs. 3 and 4) No significant difference was observed in correlation coefficients between standard SNR protocol and high SNR protocol (Table 2).

## Discussion

In this work, we have demonstrated theoretically, *in vitro* in phantom experiments and *in vivo* in human studies that increasing the SNR of a CSE-MRI acquisition by increasing voxel size improves the precision of PDFF quantification, with no impact on fat quantification accuracy. This has important implications for the use of confounder-corrected CSE-MRI methods aimed at diagnosing hepatic steatosis and for longitudinal treatment monitoring. By using acquisition protocols with higher SNR performance, more precise quantification of liver fat can be made without creating additional bias that degrades accuracy.

In the phantom study, we showed that MR parameter settings that produced higher SNR provide better test-retest repeatability in PDFF measurements made with CSE-MRI. Theoretical SNR performance, as determined by CRB analysis, demonstrated close agreement with repeatability measurements made in phantoms.

High SNR protocols, which had 2.7–3.3 times more SNR than standard SNR protocol, showed significantly improved repeatability than standard SNR protocol. Importantly, the use of a high SNR protocol did not affect the accuracy (ie: lack of bias) in either the phantom or *in vivo* clinical study.

Acquisitions that are both accurate and repeatable are necessary for quantitative imaging methods such as PDFF quantification in the liver. One practical solution for improving the estimation of true value is reducing noise in the image. Decreasing the spatial resolution and increasing the sampling interval (decreasing bandwidth) are simple and effective way to increase SNR in MRI. As hypothesized, PDFF measurements made using CSE-MRI became more robust by increasing the voxel size from ∼35–50 to ∼115∼124 mm^3^, avoiding parallel imaging (and its associated SNR penalty), and reducing bandwidth, all of which improve SNR performance.

In previous studies, the standard deviation of the difference in the PDFF measurement between 1.5T and 3T was ∼1.4 pp.^28^ Another study showed that between the different acquisition methods (breath-hold and respiratory-triggering) was ∼0.9 pp.^33^ In our study, the repeatability using standard SNR protocol (0.77–0.93 pp) was slightly better than those in previous studies, probably because the same acquisition and the same scanner were used. Using the high SNR protocol, however, repeatability of complex-fit was 0.37 pp, which means that the 95% confidence interval of Δ is less than 1 pp (−0.62 ± 0.84 pp). The high repeatability of PDFF measurements, achieved with the high SNR protocol, would enable a precise assessment of PDFF near the threshold that is required for the assessment in patients with low liver concentrations.

In the phantom study, the theoretical repeatability-SNR curve was in very good agreement with experimental measurements. These results also demonstrate a repeatability (standard deviation of Δ) of less than 0.1 pp if SNR is >70–100. However, the best repeatability *in vivo* study was 0.37 pp observed in a high SNR protocol using complex-based fit even though the SNR of the high SNR protocol were >78. This indicates that there must be other factors that affect repeatability such as biological factors like motion. Inconsistent breath-holding or other motion can be another factor that can impact the apparent accuracy and precision. Further, we also speculate that spatially varying fat concentration in the liver may be a major factor. Since perfect co-registration between two scans is not possible, subtle heterogeneity of the fat distribution in the liver would adversely impact the apparent variability of a PDFF measurement.

We applied a Monte Carlo approach to estimate the SNR of the in vivo scan.^42,46^ The standard SNR protocol usually used parallel imaging to achieve reasonable spatial resolution. Spatially varying noise amplification from the parallel imaging reconstructions, referred to as the g-factor, prevented the use of conventional SNR estimation methods where the noise is estimated using a ROI from a background region.^47^ By using a Monte Carlo approach, properly scaled and correlated noise is repeatedly added to the raw data to generate a stack of replicas. Voxel-wise estimates of the noise can be made by making voxel-wise measurements standard noise through the stack of replicas.

This study had several limitations. First, we did not evaluate the effect of decreased spatial resolution on clinical decision by radiologists, although the change in spatial resolution was relative small. In the liver MRI, we typically obtain other sequences, e.g. T_2_-weighted image and contrast-enhanced images, for the anatomical and/or morphological assessment. However, further study is necessary to determine the appropriate parameters for CSE-MRI by assessing both quantitative role as PDFF measurement and qualitative role for anatomical evaluation. Further, the use of multiple ROI’s to estimate PDFF over the liver can be used to improve the repeatability of PDFF estimates in the. However, a detailed analysis of the impact of the size and number of ROI’s on the repeatability of PDFF estimates, while relevant, is beyond the scope of this study. Another limitation is that we could not address the effect of R2 star values in the liver. During the acquisition of CSE-PDFF, six sequential echoes were obtained to correct T_2_ star effect of the liver. However, if the huge amount of iron accumulates in the liver, that may affect the repeatability of CSE-PDFF. Unfortunately, there was no large variety in R2 star values (22–52/s in right lobes of the livers), which were not large enough to study the effect of T_2_ star effect. Further study with large amount of iron in phantom/subjects are necessary to deal with this issue.

In conclusion, the use of higher SNR CSE-MRI acquisitions improves the precision of quantitative PDFF measurements in the liver, without negatively impacting the accuracy of these measurements.

## Figures and Tables

**Table 1.** MR parameters for *in vivo* study

	Scanner 1		Scanner 2	
	
	Standard SNR	High SNR	Standard SNR	High SNR
TR [ms]	14.4	12.1	15.1	12
Number of echoes	6	6	6	6
Minimum and maximum TE [ms]	1.2, 11.4	1.1, 10.5	1.2, 11.4	1.1, 10.4
Matrix	256 × 160	128 × 120	224 × 144	128 × 128
Field of view [cm]	42	42	45	45
Slice thickness [mm]	8	10	8	10
Flip angle	5°	5°	5°	5°
Number of slices^*^	32	24	32	28
Bandwidth	125	50	100	50
Partial *k_y_* acquisition	90%	80%	80%	80%
Autocalibrated parallel imaging	×2.65	-	×2.33	-
Voxel size [mm^3^]	35	115	50	124
Estimated SNR	23.7 ± 2.0	78.3 ± 6.2	52.3 ± 5.1	142.6 ± 9.8

Autocalibrated parallel imaging is expressed as the actual acceleration in acquisition time. signal-to-noise ratio (SNR) was estimated by Monte-Carlo based pseudo-multiple replica method (42). TR, repetition time; TE: echo time.

**Table 2.** Test-retest repeatability and accuracy of proton density fat fraction (PDFF) from in vivo study

	Standard SNR	High SNR	*P* value
Repeatability (standard deviation of Δ)			
*Complex-based fit*	0.77	0.37	<0.001
*Magnitude-based fit*	0.93	0.59	0.003
Accuracy (correlation coefficient) vs. MRS			
*Complex-based fit*	0.986	0.986	0.960
*Magnitude-based fit*	0.987	0.985	0.470

The difference (Δ) is the difference in PDFF between the test and retest acquisitions. Lower standard deviation of Δ implies lower variability between acquisition, ie: better repeatability. Units are given in absolute percentage points (pp), not relative percentage. *P* values are for the comparisons relative to the single region of interest (ROI) measurements from standard signal-to-noise ratio (SNR) protocol. Comparison was made using F-test for variances and t-test with Fisher r-z transformation for correlation coefficients. MRS, magnetic resonance spectroscopy.
